# Supplementary material for: Systematic Review with Meta-Analysis: Diagnostic Accuracy of Pro-C3 for Hepatic Fibrosis in Patients with Non-Alcoholic Fatty Liver Disease
Source: Biomedicines. 2021 Dec 15;9(12):1920. doi: 10.3390/biomedicines9121920 (PMC8698886; doi:10.3390/biomedicines9121920)
Supplement: Supplementary file 1 [file biomedicines-09-01920-s001.zip › Supplementary Table S1.pdf]

Supplementary Table S1: MEDLINE search strategy; run on January 2nd, 2020 and on November 1st, 2020.

1	exp Fatty Liver/	31239
2	(NAFL* or NASH*).mp.	19808
3	"non-alcoholic fatty liver disease*".mp.	15260
4	(((fatty or fat or steato*) adj3 (liver* or hepat*)) or steatohepat* or (visceral adj2 steato*)).ti,ab.	48508
5	1 or 2 or 3 or 4	60354
6	exp "sensitivity and specificity"/ or exp "mass screening"/ or "reference values"/ or "false positive reactions"/ or "false negative reactions"/ or specificit$.tw. or screening.tw. or false positive$.tw. or false negative$.tw. or accuracy.tw. or predictive value$.tw. or reference value$.tw. or roc$.tw. or likelihood ratio$.tw. or predictive value$.tw.	1925738
7	(type III pro-collagen or type III procollagen or type 3 pro-collagen or type 3 procollagen or Pro-C3 or Proc3 or P3NP or procollagen type III N-terminal peptide or PIIINP or N-terminal propeptide of type III collagen).ti,ab,kf.	1282
8	5 and 6 and 7	8
9	exp animals/ not humans/	4648880
10	8 not 9	8
