# Supplementary material for: Systematic Review with Meta-Analysis: Diagnostic Accuracy of Pro-C3 for Hepatic Fibrosis in Patients with Non-Alcoholic Fatty Liver Disease
Source: Biomedicines. 2021 Dec 15;9(12):1920. doi: 10.3390/biomedicines9121920 (PMC8698886; doi:10.3390/biomedicines9121920)
Supplement: Supplementary file 1 [file biomedicines-09-01920-s001.zip › Supplementary Table S2.pdf]

Supplementary Table S2. Additional data received from authors of primary publications

Study ID	Additional data requested?	What extra data received
**Daniels 2019** [29]	Yes	2x2 data and cut-off for Pro-C3 alone (not ADAPT panel)
**Boyle 2019** [28]	Not necessary	-
**Huber 2019** [44]	Yes	2x2 data at two suggested cut-offs (15.6 and 21.3 ng/mL)
**Luo 2018** [23]	Not necessary	-
**Nielsen, Leeming 2021** [30]	Not necessary	-
**Bril 2019** [41]	Yes	2x2 data for patients with NAFLD only, excluding the "No NAFLD" group
**Knöchel 2021** [42]	Yes	Confirmation of exclusion criteria and no overlapping patient groups with other included studies; 2x2 data at two suggested cut-offs (15.6 and 21.3 ng/mL)
**Erhardtsen 2021** [45]	Not necessary	-
