# Supplementary material for: Systematic Review with Meta-Analysis: Diagnostic Accuracy of Pro-C3 for Hepatic Fibrosis in Patients with Non-Alcoholic Fatty Liver Disease
Source: Biomedicines. 2021 Dec 15;9(12):1920. doi: 10.3390/biomedicines9121920 (PMC8698886; doi:10.3390/biomedicines9121920)
Supplement: Supplementary file 1 [file biomedicines-09-01920-s001.zip › Supplementary Table S3.pdf]

Supplementary Table S3: Incorporation of Pro-C3 into diagnostic panels for advanced fibrosis in patients with NAFLD.

Name	Panel components	Formula	Reported AUC (95% CI)
ADAPT	Age, platelets, diabetes, Pro-C3	ADAPT = exp(log10((Age x Pro-C3)/sqrt(Platelets))) + Diabetes	0.87 (0.83-0.91)(22)<br>0.85 (0.82-0.89)(21)
FIBC3	Age, BMI, T2DM, platelets, Pro-C3	FIBC3 = -5.939 + (0.053*Age) + (0.076*BMI) + (1.614*T2DM) – (0.009*platelets) + (0.071*PRO-C3)	0.85 (0.81-0.89)(21)
ABC3D	Age, BMI, platelet count < 200, Pro-C3, diabetes	Age ≫50 = 1 point, BMI ≫30 = 1 point, platelet Count ≪200 = 1 point, PRO–C3 ≫15.5 = 1 point, Diabetes = 2 points	0.83 (0.79-0.87)(21)
