# Supplementary figures and images for: Systematic Review with Meta-Analysis: Diagnostic Accuracy of Pro-C3 for Hepatic Fibrosis in Patients with Non-Alcoholic Fatty Liver Disease
Source: Biomedicines. 2021 Dec 15;9(12):1920. doi: 10.3390/biomedicines9121920 (PMC8698886; doi:10.3390/biomedicines9121920)

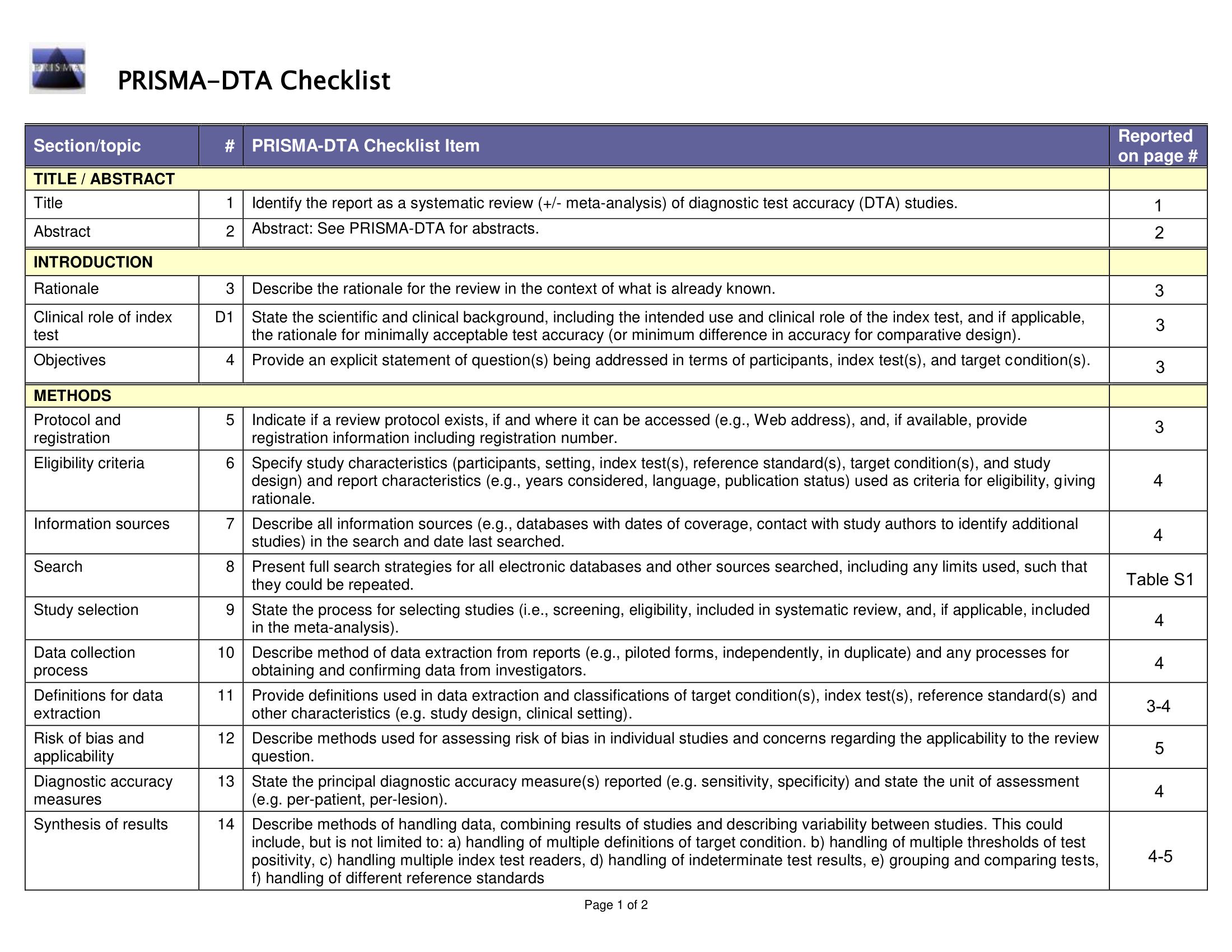

Supplement: Supplementary file 1 [file biomedicines-09-01920-s001.zip › Suppl Figure S1 PRISMA DTA Checklist.tif]
